# Rapid Prototyping in Maxillofacial Rehabilitation: A Review of Literature

**DOI:** 10.7759/cureus.28969

**Published:** 2022-09-09

**Authors:** Akansha V Bansod, Sweta G Pisulkar, Chinmayee Dahihandekar, Arushi Beri

**Affiliations:** 1 Prosthodontics, Sharad Pawar Dental College and Hospital, Datta Meghe Institute of Medical Sciences, Wardha, IND; 2 Prosthodontics and Crown and Bridge, Sharad Pawar Dental College and Hospital, Datta Meghe Institute of Medical Sciences, Wardha, IND

**Keywords:** maxillofacial prosthesis, ‎3d printing, stereolithography, rapid prototyping, maxillofacial models

## Abstract

This review focuses on fast prototyping advancements in the field of maxillofacial prosthodontics, as well as the various methods for fabricating maxillofacial prostheses. As of date, the interface and software used for processing and designing maxillofacial prostheses are costlier, atypical for the specific purpose, and only reachable to highly trained dental specialists or computer-aided design (CAD) engineers. This review is a summary of all rapid prototyping trials conducted in the mentioned context of three-dimensional (3D) printing of maxillofacial prostheses, treatment modalities, and future perspectives relating to rapid prototyping in dentistry. We performed a search of relevant articles on Google Scholar and PubMed, which yielded a total of 21 articles for full-text reviews. After excluding some articles based on the exclusion criteria, a review was conducted. This study gives a comprehensive discussion of current issues and future ideas for integrating digital technology with conventional techniques.

## Introduction and background

Rapid prototyping (RP) is an industrial revolution that has evolved hastily. People are interested in innovation in general, particularly when the eventual result can give tangible benefits. RP is a valuable tool for prosthodontic design and simulation, and it is the technology of the future. The transition from visual to the visual and tactile depiction of bodily objects ushers in a new type of collaboration known as "Touch to comprehend." The birth of the newer technology capable of directly manufacturing bodily items from graphical data generated using computer software is discussed in this article [1].

**Figure 1:**
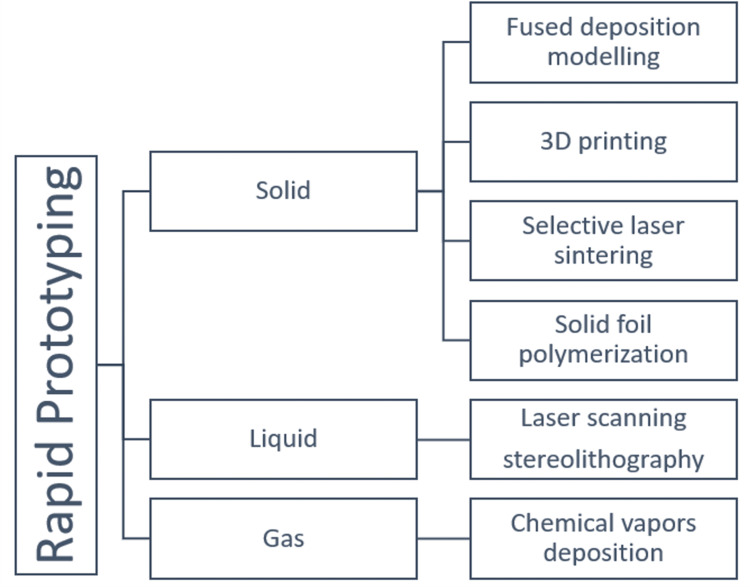
Overview of Different Types of Rapid Prototyping Used in Dentistry 3D: three dimensional

From graphical computer data, mechanical models are created and this type of computer-aided prototyping is RP. It can be done in two ways: subtractive and additive, a term that refers to a substance that is commonly utilized. The additive manufacturing (AM) method varied from old-style subtractive manufacturing principles and is now used in a variety of fields, including personalized medicine, aerospace, and dental specialty. This method of manufacturing allows for the rapid manufacture of custom-based complicated parts, making it a viable option for self-growing robot development [2]. Inkjet printing, fused deposition modeling (FDM), stereolithography, selective laser sintering, photopolymer jetting, and printing with the precipitate binder are examples of additive manufacturing technologies. RP is broadly categorized into additive and subtractive technology. An overview of various types of RP used is mentioned in Figure 1 [3].

## Review

Material and methods

**Figure 2:**
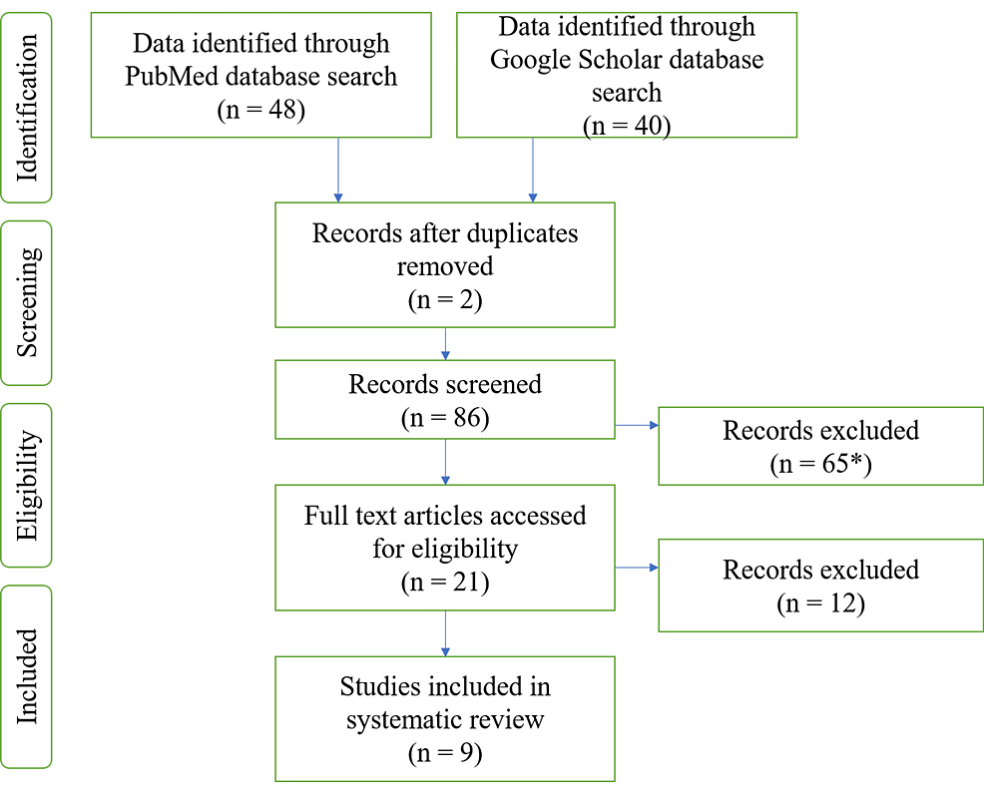
PRISMA Flowchart for the Studies Included in the Systematic Review *The articles excluded after reading the title PRISMA: Preferred Reporting Items for Systematic Reviews and Meta-Analyses

The protocol for this review was registered with the International Prospective Register of Systematic Reviews (PROSPERO) with the registration number CRD42021251023. We followed the Preferred Reporting Items for Systematic Reviews and Meta-Analyses (PRISMA) guidelines to conduct this review. The PRISMA flowchart for the selected studies is given in Figure 2.

The assessment was based on the population, intervention, control, and outcomes (PICO) study criteria. The electronic search on the Google Scholar database provided a total of nine articles that were considered potentially relevant. The texts found using the “[AND] & [OR]” Boolean operators in between the search words "Rapid Prototyping", "Maxillofacial Prostheses", "3D Printing", "Stereolithography", "Dentistry", "Dentofacial Prostheses" were 88.

In the second phase of article selection, all articles selected needed to be in the English language. A total of 65 articles were excluded after reading the title, and all duplicate articles were excluded. A total of 21 articles were selected for the systematic review. Of these 21 articles, after reading the complete text, the most relevant nine articles were selected for the systematic review.

The search strategy showing article search through PubMed database search is summarized in Table 1 

**Table 1:** Flowchart Showing Article Search Through PubMed Database The keywords used were  "Rapid Prototyping", "Maxillofacial Prostheses", "3D Printing", "Stereolithography", "Dentistry", and "Dentofacial Prostheses"  using the “[AND] & [OR]” Boolean operators in between the search words

Search results combined after screening the PubMed database	48
Articles not in the English language excluded	1
Articles excluded after reading the title	24
Duplicate articles excluded	2
15 articles were searched for full texts	9 articles were excluded, and a total of 6 articles were selected for the review

The search strategy showing article search through Google Scholar database search is summarized in Table 2. 

**Table 2:** Flowchart Showing Article Search through Google Scholar Database The keywords used were  "Rapid Prototyping", "Maxillofacial Prostheses", "3D Printing", "Stereolithography", "Dentistry", and "Dentofacial Prostheses"  using the “[AND] & [OR]” Boolean operators in between the search words

Search results combined after screening the Google Scholar database	40
Articles not in the English language excluded	1
Articles excluded after reading the title	21
Duplicate articles excluded	2
16 articles were searched for full texts	13 articles were excluded, and a total of 3 articles were selected for the review

Discussion

The use of 3D printing technology in several aspects of modern dental medicine has permitted the fabrication of sophisticated prosthodontic, surgical, and orthodontic devices that require the molding materials to be flexible and abrasion-resistant (Table 3). Different materials, e.g., composites, polymers, ceramics, and metallic blends, are employed for additive manufacturing [4]. Innovations in molding materials and forming procedures have improved RP techniques to the point where this technology is used for more than just prototyping; it is also used to reproduce real functional elements [5]. The feasibility of this technique is increasing in a variety of dental practice fields, including oro-maxillofacial surgery and prosthesis, the production of surgical guides or physical models in dental implant therapies, and prosthodontics [6-10].

**Table 3:** Applications of Rapid Prototyping in Dentistry

Applications of rapid prototyping in dentistry	
Prosthodontics	Wax pattern fabrication,direct prosthesis milling, 3D graphic data for complete denture fabrication, fabrication of maxillofacial prostheses and obturators, guided Implant surgeries, training and research
Endodontics	3D visualization of complex canals, accurate diagnosis and treatment planning, training and research
Orthodontics	Diagnosis and treatment planning, fabrication of appliances, aligners, lingualized orthodontics, 3D models for orthognathic surgery
Oral and Maxillofacial Surgery	Fabrication of surgical guides, assessment of cases

Dental prostheses such as crowns, removable and fixed partial dentures (RPDs and FPDs), and metal copings can also be planned, manufactured, and developed using RP techniques. This technique saves time and intervention in traditional prosthesis fabrication and also aids in the elimination of any flaws caused by human skills. To create frameworks for cast partial dentures, digital dental surveying and RP-produced patterns can be used [11]. Furthermore, RP also reduces the amount of extra-oral time required for autogenous tooth transplantation. The dental practice has profited from RP in the accurate reconstruction of maxillofacial defects as well as in osteogenic distraction with promising results [12-15]. Further discussion is mainly focused on the reconstruction of maxillofacial defects and prosthesis fabrication using rapid prototyping.

3D Printing Technique

Data from cone beam computed tomography (CBCT) and optical scanners (IOS) pictures form the basis of 3D printing technology. This information is then transformed into a standard tessellation language file (STL), which can then be imported into 3D modeling software and altered to match the clinician's manufacturing requirements. Clinicians then upload the files to their preferred printer after making these changes. The prosthesis might be printed directly or a mold could be made for more traditional silicone manufacture [12].

Stereolithography (SLA), selective laser sintering (SLS), inkjet-based systems, and fused deposition modeling (FDM) are the most frequently used technologies in dental practice. Digital light processing (DLP), SLA, and material jetting (MJ) are the three most prevalent categories of 3D printing used in dentistry. On top of the building printer platform, the machine uses additive fabrication processes to create a prosthesis. Prostheses can be printed using a variety of materials, including ceramic, metal, and thermoplastic resin. Following manufacture, post-manufacturing operations are carried out to verify that the product is free of flaws and correctly processed; the scope of these steps varies according to the printer type and the material being used. It should be highlighted that the correctness and precision of each printer type are greatly indicative of the quality of the printer, the technology used, the materials, the settings in the software, and the post-manufacturing refining process. The interconnection of all of the features has a greater impact on overall quality than the differences in production processes like SLA, DLP, and MJ [15-17].

Use of RP Techniques in Dental and Facial Prosthetics

RP techniques are now observed as a promising and satisfactory alternative for the fabrication and manufacture of dental prostheses. Molding of a dental (facial) prosthesis and metal casting mold (shell) is now performed in a short period of time. Using an incremental printing method, 3D printing creates ceramic casting molds for metal casting. Many time-consuming steps and labor-intensive work of the traditional investment casting technique are eliminated with RP techniques. The technique also eliminates the need for wax and core tooling design and manufacturing, wax and core molding, wax assembly, shell dipping and drying, and wax elimination [4-6].

Facial Prosthesis Mold

**Figure 3:**
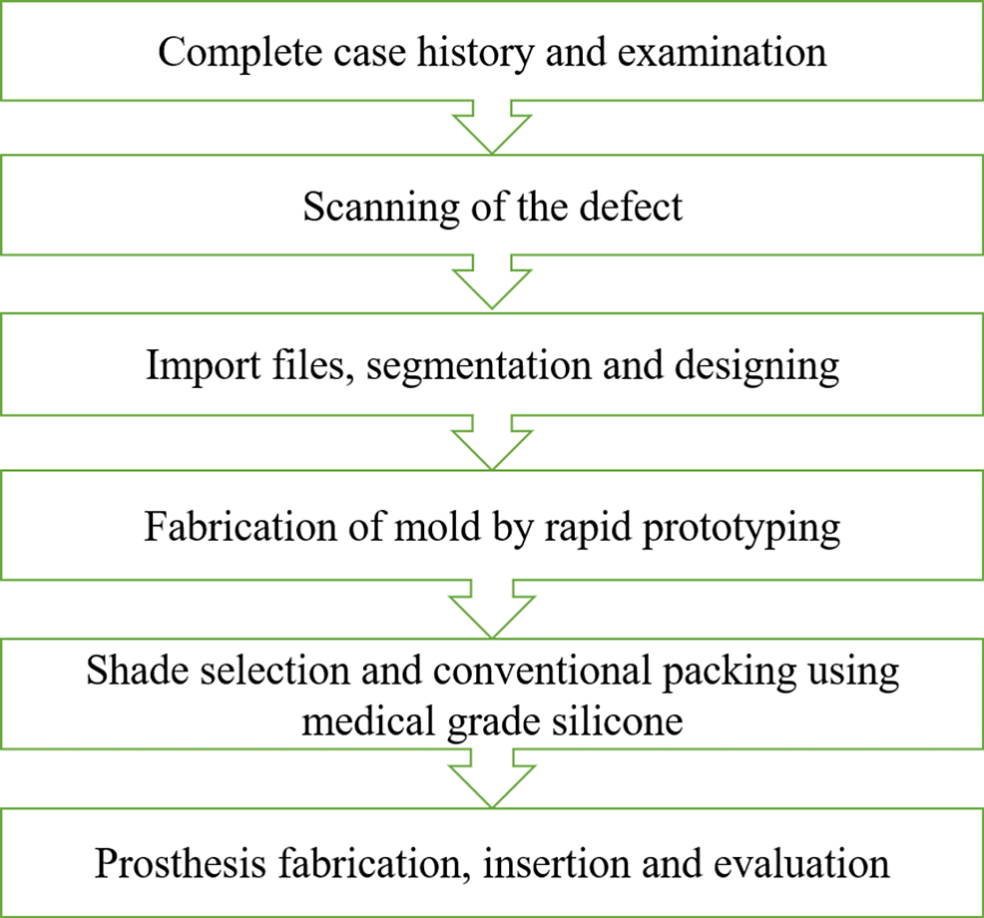
Hybrid Protocol for the Workflow of Maxillofacial Prostheses Combining the conventional and digital techniques for the fabrication of maxillofacial prostheses, a hybrid protocol has been formulated

Over the last decade, RP techniques have been used successfully to fabricate facial prostheses. Although pattern fabrication with the aid of RP was a feasible procedure, the traditional flasking and investing procedures were still required to make the actual prosthesis. Using a mold would eliminate the need for traditional flasking and investment procedures, as well as shorten the process of creating the prosthesis. Furthermore, the generated resin mold can be kept because it is long-lasting and allows for multiple pourings [7]. Combining the digital and conventional techniques, a hybrid protocol for the fabrication of maxillofacial prosthesis has been described in Figure 3. A comparative evaluation of different techniques used for maxillofacial prostheses fabrication is done in Table 4.

**Table 4:** Comparative Evaluation of Different Techniques used for Maxillofacial Prostheses Fabrication.

	Workflow	Clinical efficacy	Time	Cost-effectiveness	Edge quality and marginal adaptation	Aesthetic outcomes	Material characteristics
CONVENTIONAL FABRICATION	Manual Impression making and multiple try-ins	Several complex steps, labor-intensive	Time-consuming	Cheaper, compared to digital technique	Good	The Patient relies on the skills of the Prosthodontist	Medical grade silicone
HYBRID	3D capture of facial topography	Excellent; contactless Semi-automated	Less time-consuming compared to Conventional fabrication	Cuts off the additional digital fabrication costs	Acceptable	Acceptable	Medical grade silicone
DIGITAL FABRICATION	3D capture of facial topography	Excellent; sometimes challenging and prone to errors	Minimal time required	Expensive	Comparatively low	Acceptable	No material is clinically approved for direct fabrication

Conventional Workflow: Choosing an appropriate impression technique and material (irreversible hydrocolloids or elastic silicones are the most commonly utilized impression materials) based on the type of defect, the size, and the presence of undercuts in the affected part, and a custom tray is important. To retrieve the impression without causing any damage to tissue in the surrounding, some anatomic undercuts are blocked. The gypsum cast is obtained when the impression is poured, and a wax pattern of the anatomic portion to be replaced is made up. The wax is carved to reproduce the defect's natural morphological details, followed by a try-in step of the prosthesis wax-up with the equivalent maxillofacial prosthesis [16].

Digital Workflow: The final prostheses are created using rapid prototyping, specifically additive manufacturing. Maxillofacial prostheses are fabricated indirectly by procurement of a mold or model of the prosthesis, followed by the traditional workflow for part processing, or directly manufacturing with the help of 3D printing with adequate material, depending on the anticipated digital workflow and the material being used (e.g., acrylic resins, silicone-based elastomers, and others) [17].

Recent Advances

3D Bioprinting, a combination of 3D printing and tissue engineering is a rapidly expanding technology in the field of regenerative medicine for autograft production. Biomaterials, bioactive substances, and even cells that are carefully positioned and with spatial control can be 3D printed to reconstruct human tissues and organs that can imitate their native counterparts in terms of both shape and function. This process is known as 3D bioprinting [5,7]. It's the result of combining 3D printing with tissue engineering. Tissue engineering is a field of regenerative medicine that tries to construct an autologous graft using the patient's own cells.

Additive manufacturing technology, such as 3D printing, is now frequently used to improve the aesthetics of maxillofacial prostheses with precise 3D fabrication. It uses CAD software to create complicated facial shapes, which is then followed by layer-by-layer material deposition to create 3D objects. It can make not just complex craniofacial analogs but also can manufacture prototypes for osteotomy guides, bone grafts, and occlusal splints to be used intraoperatively, which increases efficiency and makes surgery easier. However, creating indistinguishable maxillofacial prostheses continues to be a challenge [7,12].

## Conclusions

It is clear that 3D RP is an important tool for creating maxillofacial prostheses and 3D bioprinting is a boon in creating complex tissues and organs, such as muscular tissue, and for using biomaterials to manufacture and develop the extra segments. RP techniques are currently playing a larger role and will play an important role in prosthodontics the dominant digital fabrication technologies. There are, however, important problems in the process of innovation that must be addressed. The majority of currently available tissue products closely resemble genuine tissues and have emerged with a focus on tissue removal and multicellular systems. The first aspects of making biopolymer manufacture and obtaining standards should be in sync with the creative cycle. Learning and utilizing the 3D printing technique and achieving the standards requires immediate assistance. In the forthcoming era, clinicians should assume that 3D printing technology will have applications in a wide array of dentistry fields, especially maxillofacial rehabilitation.
